# Schema Therapy for Individuals With Subthreshold Autistic Traits and Recurrent Depressive Disorder: A Case Study

**DOI:** 10.7759/cureus.87357

**Published:** 2025-07-05

**Authors:** Tsukasa Murakami, Fumiyo Oshima, Takafumi Morimoto, Hiroki Annaka

**Affiliations:** 1 Faculty of Rehabilitation Sciences, Niigata University of Health and Welfare, Niigata, JPN; 2 Research Center for Child Mental Development, Chiba University, Chiba, JPN; 3 Department of Occupational Therapy, School of Health Sciences, Sapporo Medical University, Sapporo, JPN

**Keywords:** autism spectrum disorder, autistic traits, depression, schema therapy, trauma

## Abstract

Schema therapy (ST) is a therapeutic approach to address chronic and complex psychological difficulties. ST is also used to treat mental health problems that individuals on the autism spectrum have. However, no practical reports exist on its application to cases involving subthreshold autistic traits with associated psychological difficulties. This report describes schema therapy for a woman with subthreshold autistic traits and raised in an abusive environment. The intervention was based on schema therapy for individuals on the autism spectrum and consisted of a total of 42 sessions. The effects of schema therapy remained effective at an eight-month follow-up. This practice suggests that the promotion of understanding of autistic traits and experiential techniques are helpful in schema therapy for cases with subthreshold autistic traits.

## Introduction

Autism spectrum disorder (ASD) is a neurodevelopmental condition characterized by impairments in communication, interpersonal relationships, cognition, and sensory perception [1]. Prevalence estimates have increased over the years [2], with particular attention being paid to supporting women in adulthood who have difficulties due to autistic spectrum features that are mild or below the diagnostic threshold [3,4]. Additionally, individuals on the autism spectrum are at heightened risk of interpersonal trauma, such as abuse and bullying [5], which may lead to further complications, such as complex post-traumatic stress disorder (PTSD) or personality disorders. However, in cases of mild or below diagnostic threshold ASD in women, there is concern that ASD may be overlooked during consultations for comorbid psychiatric symptoms, potentially resulting in inappropriate treatments [6,7]. It is believed that women on the autism spectrum have unique characteristics, such as having different interests and concerns than men and being prone to “camouflage behavior” to fit in with their surroundings in social situations [6]. As a result, people around them may not be aware of the autistic traits and may diagnose it only with superficial symptoms such as depression and anxiety [6]. In addition, individuals with subthreshold autistic traits are at similarly high psychiatric risk as those with threshold autistic traits [7]. Subthreshold autistic traits refer to individuals who partially meet the diagnostic criteria for ASD [3]. These individuals may face challenges, such as difficulties integrating into groups and forming relationships [3]. Additionally, despite having normal or even above-average intelligence, deficits in communication and social interaction can significantly impact their academic performance [3]. Therefore, providing appropriate mental health support to individuals with subthreshold autistic traits is essential, even if they do not meet the full diagnostic criteria.

Schema therapy (ST) is a therapeutic approach to address chronic and complex psychological difficulties [8]. This integrative psychotherapy incorporates elements of cognitive behavioral therapy, attachment theory, gestalt therapy, object relations theory, and constructivism. Research has demonstrated its effectiveness for a wide range of conditions, including personality disorders, obsessive-compulsive and anxiety disorders, and PTSD [9,10]. At the core of ST are two key concepts: early maladaptive schemas (EMS) and schema modes (SM). EMS develops when core emotional needs remain unmet due to the interaction between an individual’s temperament and adverse early environmental factors. SM refers to the currently active schemas and their associated functions in the individual’s present state [8]. The treatment process in ST generally consists of the assessment and education phase and the change phase [8]. During the assessment and education phase, therapists work to identify the client’s schemas and modes while helping them draw connections between their current difficulties and early life experiences. The change phase includes cognitive strategies (e.g., evaluating the validity of schemas and modes), behavioral methods (e.g., role-playing and exposure), and experiential approaches (e.g., imagery rescripting and chair-work exercises) [11].

Adults on the autism spectrum are more likely to have chronic mental health problems, and scoping review results report that schema therapy may be effective [12]. However, research on schema therapy for adults on the autism spectrum is scarce; more knowledge needs to be accumulated [12]. Regarding the application of schema therapy for adults on the autism spectrum, reports by Oshima et al. [13], Oshima et al. [14], and Vuijk et al. [15] have been published. The ST protocol for adults on the autism spectrum, as described by Oshima et al. [13], comprises three phases: the psychoeducational and mode conceptualization phase, the schema mode change phase, and the behavioral pattern-breaking intervention phase. The psychoeducational and mode conceptualization phase includes psychoeducational interventions focusing on autistic traits and differences in life experiences between those who are neurotypical and adults on the autism spectrum. The schema mode change phase and the behavioral pattern-breaking intervention phase correspond to the change phase described by Young et al. [8]. However, these studies were conducted with individuals diagnosed with ASD, and there are no reports of schema therapy for individuals with subthreshold autistic traits [13-15]. Considering that subthreshold cases often present with complex psychosocial difficulties [3,4], ST may also be beneficial in cases involving subthreshold autistic traits with associated psychological difficulties. Therefore, it is highly significant to examine the effectiveness of schema therapy for this population. This report aims to examine the significance and considerations of schema therapy applied to a client with subthreshold autistic traits and recurrent depression, referencing the protocol for adults on the autism spectrum by Oshima et al. [13].

## Case presentation

The client was a 39-year-old woman diagnosed with recurrent depressive disorder. She had a history of panic disorder and eating disorder diagnoses. At the client’s request, no pharmacological treatment was implemented. In the past, the client had utilized psychiatric daycare services but eventually stopped attending due to interpersonal difficulties. Specifically, she displayed overly adaptive behavior in interactions with other participants but eventually expressed anger toward those with whom she had developed closer relationships, leading to her voluntary withdrawal. Observations during her daycare use revealed characteristics such as strong adherence to rules and competition, difficulty monitoring emotions and fatigue, discomfort with unpredictable situations, misinterpretation of implicit intentions, sensory peculiarities, and challenges with unstructured environments. The client thought that these difficulties were “traumatic from past experiences” and requested counseling.

Family history

When the client was one or two years old, her parents divorced. Therefore, she lived with her mother in a separate house on her maternal grandparents’ property. The client’s mother, diagnosed with schizophrenia, has experienced repeated hospitalizations in psychiatric facilities. Since the client’s early childhood, her mother has faced significant parenting challenges due to her mental health symptoms and frequent overdoses. These difficulties manifested in various ways, including indifference to the client’s emotions and neglect of responsibilities, such as failing to prepare the client’s school lunches and leaving the client alone at night to go out. At the time ST began, the mother was residing in a care facility for the elderly but was occasionally hospitalized when her condition worsened. Although the client wanted to distance herself from her mother, she was voluntarily involved with her mother, taking charge of all hospitalization and institutionalization procedures.

The client’s grandparents ran a business from their home. The client viewed her grandfather as a symbol of security. However, he often told her, “You are a pitiable child,” citing her mother’s mental illness and the absence of her father. In addition, the grandparents tried to care for the client but did not listen to her feelings very often.

Case history

Since early elementary school, the client has struggled with group conversations, expressing her feelings, and increased anxiety in new environments, resulting in frequent early dismissals and absences from school. In middle school, the client wanted to attend a private school, but her grandfather denied the request, citing her mother’s health problems. This led to frustration and resentment, causing the client to act aggressively at home. She enrolled in a local high school but dropped out after her first year and later enrolled in a correspondence high school. During this time, she frequently avoided returning home, spent time in the entertainment district, and engaged in relationships with several men and sexually deviant behaviors. After graduating from high school, the client began experiencing episodes of hyperventilation, which prompted her to seek medical attention. The client also developed significant anorexic tendencies. The client was then transferred to several hospitals for treatment.

The client spent 1-2 years helping in the family business and working part-time as a bookstore clerk and bar hostess, when she met and married her husband. She has not worked since her marriage and now lives with her husband and a pet rabbit that has become the center of her daily life. There was a time when the client could not spend time alone at home and spent time in her husband’s company. However, their relationship is unstable, as her husband alternates between being supportive and dismissive or aggressive. Their emotional conflicts have occasionally escalated to the point of police intervention during marital disputes.

The client’s primary challenges can be summarized as follows: inability to go out alone, difficulty maintaining emotionally stable relationships, a codependent marital relationship, and a codependent mother-daughter relationship.

Psychological assessment

The client’s score on the Japanese version of the Autism Spectrum Quotient exceeded the cutoff score, suggesting significant autistic traits. Additionally, results from the Japanese version of the Adolescent/Adult Sensory Profile highlighted pronounced tendencies toward sensory hypersensitivity and sensory avoidance. The Wechsler Adult Intelligence Scale (third edition) results showed a full-scale IQ (FIQ) of 115, with a score of 105 in verbal comprehension, 112 in perceptual organization, 107 in working memory, and 137 in processing speed.

Treatment

ST was conducted every other week between 2022 and 2024, totaling 42 sessions including follow-ups. Each session lasted 45 minutes. The treatment was provided as part of insurance-covered medical services. The therapist completed 40 hours of schema therapy training and conducted sessions while receiving supervision approximately once a month. The structure of each session is shown in Table 1.

**Table 1:** Details for schema therapy for adults on the autism spectrum and additional plans ASD: autism spectrum disorder, EMS: early maladaptive schemas, SM: schema modes

Phase	Protocol of the schema therapy for adults on the autism spectrum by Oshima et al. [13]	Additional plans for this case
Psychoeducational and mode conceptualization phase (session 1-15)	Understanding ASD and the differences in life between neurotypicals and adults on the autism spectrum; understanding EMS and SM; understanding core emotional needs; understanding symptoms using schema theory	Did not use the term “ASD” and consistently described them as “autistic traits” for the client; developed an understanding of specific traits, such as “sensory oversensitivity,” through experience; presented case formulations visually
Schema mode change phase (session 16-30)	Understanding maladaptive coping mode; experience to meet core emotional needs; developing their healthy adult mode	Explain the purpose and process of implementing techniques such as imagery rescripting in advance using materials
Behavioral pattern-breaking intervention phase (session 31-42)	Problem-solving using healthy adult mode - handle conflict situations	Making lifestyle adjustments in accordance with the client’s traits

Intervention

Psychoeducational and Mode Conceptualization Phase (Session 1-15)

Psychoeducation on the concepts of ST was provided, along with education on autistic traits and attention deficit hyperactivity disorder (ADHD) traits. The client was guided to develop a cognitive understanding that their difficulties in daily life stemmed from a mismatch between their traits and the surrounding environment. This phase focused on introducing the concepts of ST, fostering an understanding of the traits, and gathering her developmental history.

The client showed a limited understanding of traits and found it challenging to accept terms such as “autistic traits.” However, she agreed with specific characteristics such as difficulty verbalizing her feelings, hypersensitivity, being very particular, poor self-monitoring, interpreting implied meanings without thinking about what is meant, and sometimes acting impulsively. Regarding her hypersensitivity, the client noticed a reduction in discomfort when the lighting in the counseling room was adjusted. Additionally, when learning about camouflage, the client showed strong interest, recognizing its alignment with her experiences. Also, the overall framework of ST and case formulation were visually presented to address the client’s difficulty with predictability. The client positively accepted these considerations. The developmental history revealed that the client experienced emotional harm and lacked a safe environment at both home and school. After deepening the understanding of the client’s background and traits, we identified unmet core emotional needs: secure attachment to others, freedom to express emotions, and a sense of autonomy, competence, and identity.

**Figure 1:**
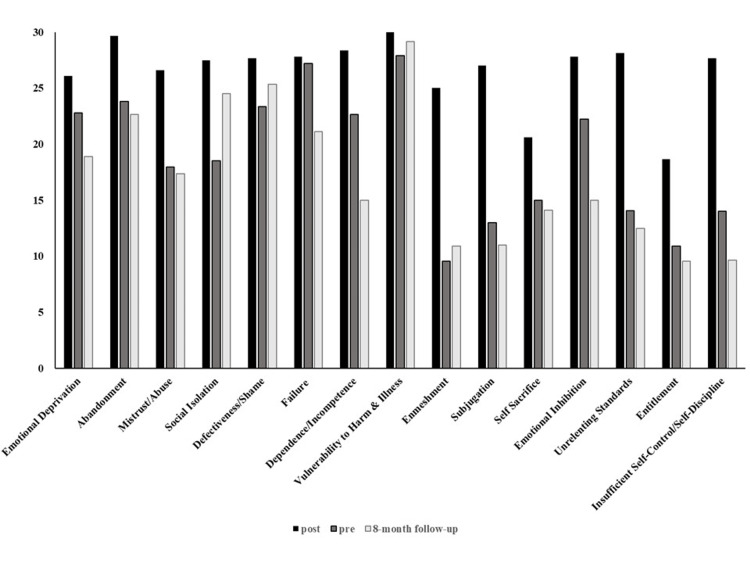
Change in YSQ-SF scores before and after schema therapy implementation YSQ-SF: Young Schema Questionnaire-Short Form Japanese version

Subsequently, the Young Schema Questionnaire-Short Form Japanese version (YSQ-SF) [16] was administered (Figure 1). The YSQ-SF can measure 15 types of EMS: emotional deprivation, abandonment, mistrust/abuse, social isolation, defectiveness/shame, failure, dependence/incompetence, vulnerability to harm or illness, enmeshment, subjugation, self-sacrifice, emotional inhibition, unrelenting standards, entitlement, and insufficient self-control and self-discipline. Through discussions, the client understood that her SM and EMS were shaped by adverse experiences and a lack of support suited to their unique characteristics. The goals of ST were identified as reducing anxiety, fear, and distrust associated with acting, expanding the range of self-sufficient activities and actions in daily life, and developing sustainable interpersonal relationships. The intervention focused on the following modes: the abandoned/abused child mode (characterized by heightened feelings of shame and distrust of others; the vulnerable child mode), the dependent child mode (marked by a lack of confidence and increased worry; the vulnerable child mode), the compliant surrender mode (overly accommodating to others), and the demanding parent mode (demanding that significant others behave in a way that meets her need).

Schema Mode Change Phase (Session 16-30)

The intervention aimed to process emotions and cognitions related to childhood experiences through experiential techniques. Mainly, imagery work on safe places and imagery rescripting were used. In the work on safe places, the client imagined “a white fluffy bed in a room.” The client liked this image and used it in her daily life. The client also sought out reassuring pictures and objects. A picture of her pet rabbit and a comfortable blanket helped her feel safe and secure.

After this process, imagery rescripting was introduced. The intervention began by addressing an incident in which the client was told that their mother had given away an item they cherished without any explanation. Gradually, the focus shifted to addressing experiences that had caused the client deeper emotional pain. Table 2 shows examples of imagery rescripting. During an imagery rescripting, where the therapist explained to the grandfather that he needed to explain situations to the client carefully and that the client struggled with verbalizing their thoughts and emotions and required support, the client felt deeply understood and began to cry. Through these experiences, the client gradually became aware that they had been emotionally over-involved with their mother and that their grandfather’s parenting approach had not been suited to their characteristics.

**Table 2:** Example of imagery rescripting (schema mode change phase (session 16-30))

Scene	Scenario	Client response
When the client was in elementary school, she was told that her mother had given away her prized possessions to someone else without explanation.	The therapist explains to the client’s mother the client’s traits, the importance of respecting the client, and the child’s rights.	She said, “I was glad you spoke up for my rights.”
When the client was in the early grades of elementary school, her mother was overmedicated and unstable at home, and she was worried because she did not know what to do.	The therapist explains to the client’s mother that the client will be taken to a protective and safe environment. The client then spends time in that environment, feeling safe.	She said, “At first, I remembered the fear, but then I had a sense of being protected.”
When the client was in elementary school, her mother disappeared from their home without explanation. At that time, she expressed his feelings of anxiety to her grandfather, but the grandfather did not listen to her feelings.	The therapist explains her autistic traits to the client’s grandfather and tells him to change the way he relates to the client.	She said, “My grandfather understood that I was in trouble,” and burst into tears.

Behavioral Pattern-Breaking Intervention Phase (Session 31-42)

The intervention focused on fostering the healthy adult mode. During this phase, we explored meaningful behaviors for the client, delved into healthy adult mode, and discussed strategies to sustain it. To support the client in expanding her living area, we planned opportunities for her to go out alone. Although the client could not take the subway alone, she could use a taxi independently and ride the streetcar when accompanied by her husband.

Changes also began to be observed in the client’s relationship with her mother and husband. First, her mother was becoming increasingly unwell due to pneumonia. Therefore, the client decided and acted to support her until her end-of-life care while maintaining a sense of distance. During the sessions, consultations and role-plays on encouraging her husband to see a doctor were also conducted regarding her relationship with her husband. Around this time, the client exhibited notable behavioral changes, which led to the conclusion of ST.

A follow-up interview was conducted eight months later. At that time, the client reported that her mother had died three months after the intervention ended, followed by the death of her rabbit seven months later. During the interview, while the client expressed her sadness at the loss of the rabbit, she reflected on the intervention period and stated that she had come to terms with her feelings toward her mother. They also expressed a positive intention to live their own lives in the future. The YSQ-SF score showed an overall improvement from the start of the intervention; however, some worsening was observed at follow-up.

## Discussion

ST was tried and validated with a client who has subthreshold autistic traits and has grown up in an abusive environment. The client had difficulties related to autistic traits, such as sensory idiosyncrasies, difficulty with imagination, and difficulty with situations that were unpredictable and lacking in perspective. The client also had difficulty adjusting to groups, and while she was not comfortable interacting with her peers, she preferred to interact with older people. In addition, the client showed a strong attachment to his pet rabbit, which provided psychological support for the client. These characteristics are considered to be those found in adults with subthreshold to mild ASD [3]. Moreover, clients may have had attachment issues due to the effects of an abusive environment; it has been suggested that individuals on the autism spectrum are more prone to attachment issues [17].

Test results have also yielded findings that suggest that the client may be in the diagnostic range of ASD. However, clients tended to use camouflaging behaviors routinely. Therefore, the physician was unable to identify the client’s autistic traits in the examination setting and was unable to make a diagnosis. The client was unaware of autistic traits and lived a life without being aware of them. As a result, the client had problems with interpersonal relationships and difficulty controlling her emotions, but missed opportunities to receive treatment for these issues. ASD in women and autistic traits of borderline personality disorder and PTSD tend to be underdiagnosed [4,6,18]. Given these backgrounds, it is important to understand individuals in terms of personality, autistic traits, and environment.

Interventions for this case were conducted based on the protocol for ST for adults on the autism spectrum [13]. In the first phase, the intervention focused on autistic traits and differences in life experiences between those who are neurotypical and adults on the autism spectrum. It has been suggested that such a program to promote self-understanding contributes to improved mental health and increased self-awareness [19]. On the other hand, some adults on the autism spectrum resist the diagnostic name ASD [20]. The client initially showed strong resistance to the term “autistic traits” and seemed to have difficulty accepting her traits. However, the client better understood her traits by discussing specific personal traits and experiences, such as sensory sensitivity, communication traits, and camouflaging behaviors. This suggests that when clients reject the diagnosis of ASD or the term “autistic traits,” it is important to promote self-understanding through specific traits and experiences rather than directly using these terms. In addition, the experience of being aware of and taking care of individual traits is limited reparenting in ST, which may be an important factor for clients with autistic traits. Schema therapy may also be more useful for clients with a limited understanding of their own traits, as it makes it easier to examine how these characteristics impact their current state.

In the second phase, experiential techniques were mainly used. Experiential techniques have been suggested to be helpful in individuals on the autism spectrum [15], and we inferred that they are equally valuable for individuals with subthreshold autistic traits. These results suggest that the ST protocol for adults on the autism spectrum may also be helpful for clients with subthreshold autistic traits. However, this report is a case report based on a single case. Therefore, it is necessary to verify the effectiveness of schema therapy for clients with subthreshold autistic traits in the future. Additionally, this report evaluates the effectiveness of ST solely through the YSQ-SF. It is necessary to examine the efficacy of other aspects, such as the patient’s quality of life and subjective well-being.

## Conclusions

This report described ST for a woman with subthreshold autistic traits who was raised in an abusive environment. The intervention included psychoeducation for autistic traits and ST and experiential techniques such as imagery rescripting. The intervention results suggest that promoting self-understanding and imagery rescripting are important components of ST for adults with subthreshold autistic traits. However, this report evaluates the effect of schema therapy on an individual with subthreshold autistic traits in one case using only the YSQ-SF. Therefore, further studies with larger sample sizes are needed to clarify the effectiveness of ST for adults with subthreshold autistic traits.
